# A paleo-perspective on West Antarctic Ice Sheet retreat

**DOI:** 10.1038/s41598-022-22450-3

**Published:** 2022-10-21

**Authors:** Philip J. Bart, Matthew Kratochvil

**Affiliations:** Department of Geology and Geophysics, Louisiana State University, Baton Rouge, LA 70803 USA

**Keywords:** Climate sciences, Climate change, Cryospheric science, Palaeoclimate

## Abstract

Geological records of ice sheet collapse can provide perspective on the ongoing retreat of grounded and floating ice. An abrupt retreat of the West Antarctic Ice Sheet (WAIS) that occurred during the early deglaciation is well recorded on the eastern Ross Sea continental shelf. There, an ice shelf breakup at 12.3 ± 0.6 cal. (calibrated) kyr BP caused accelerated ice-mass loss from the Bindschadler Ice Stream (BIS). The accelerated mass loss led to a significant negative mass balance that re-organized WAIS flow across the central and eastern Ross Sea. By ~ 11.5 ± 0.3 cal kyr BP, dynamic thinning of grounded ice triggered a retreat that opened a ~ 200-km grounding-line embayment on the Whales Deep Basin (WDB) middle continental shelf. Here, we reconstruct the pattern, duration and rate of retreat from a backstepping succession of small-scale grounding-zone ridges that formed on the embayment’s eastern flank. We used two end-member paleo-sediment fluxes, i.e., accumulation rates, to convert the cumulative sediment volumes of the ridge field to elapsed time for measured distances of grounding-line retreat. The end-members fluxes correspond to deposition rates for buttressed and unbuttressed ice stream flow. Both scenarios require sustained rapid retreat that exceeded several centuries. Grounding-line retreat is estimated to have averaged between ~ 100 ± 32 and ~ 700 ± 79 ma^−1^. The evidence favors the latter scenario because iceberg furrows that cross cut the ridges in deep water require weakly buttressed flow as the embayment opened. In comparison with the modern grounding-zone dynamics, this paleo-perspective provides confidence in model projections that a large-scale sustained contraction of grounded ice is underway in several Pacific-Ocean sectors of the WAIS.

## Introduction

Glaciers in the Amundsen Sea sector of West Antarctica are speeding up and grounding lines retreating as their ice shelves thin^1^. This raises concern that an unstable collapse may be underway^2–4^. The term collapse is not precisely defined but is generally used to describe a significant contraction in the extent and volume of grounded ice within a relatively short time. In the Bellingshausen-Sea sector, 65% of WAIS grounding lines have retreated between 1990 and 2015^5^. These dynamics are at least partly driven by upwelled circumpolar deep water that advects onto the continental shelf and melts the ice sheet’s marine margins. Since 1979, discharge from fast-flowing WAIS glacial systems has contributed nearly 7 mm of sea-level equivalent volume to global sea level rise^6^. Recent modeling studies have projected that Pine Island Glacier losses could exceed 100 Gt/yr^−1^ within 20 years^3^. Model simulations project that a transition to an even more rapid retreat could occur within 200–900 years^2^. These projections all rely on two tacit assumptions: firstly, that the current rapid contractions are anomalously fast and secondly, that the fast rates will be sustained for several centuries. The assumptions are important to justify because changes to these large-scale glacial systems are probably complex and some studies have shown that feedbacks such as isostatic rebound^7^, grounding zone sedimentation^8^ or sea-level fall^9^ may halt and/or even reverse rapid flow and retreat.

**Figure 1:**
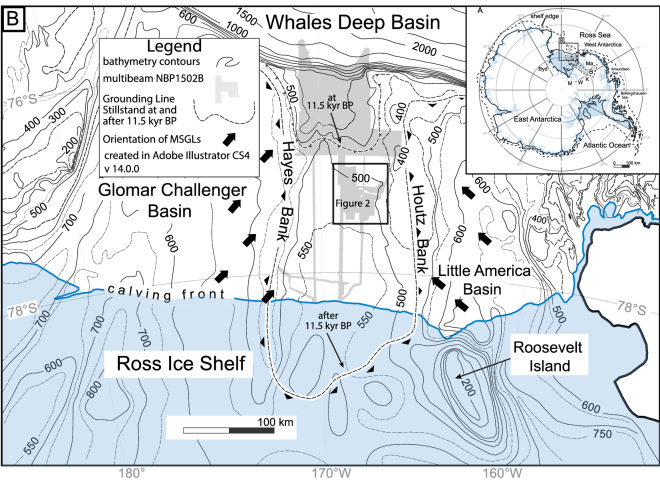
(**A**) Map of Antarctica showing the locations of the Whillans (W), Kamb (K), Bindschadler (B) and MacAyeal (M) ice streams. RIS = Ross Ice Shelf. GL = modern grounding line^6,8^. (**B**) Bathymetry contours from Davey and Nitsche^14^ for eastern Ross Sea north of the Ross Ice Shelf calving front. The gray shade shows the location of multibeam and sonar data acquired during NBP1502B^16^. The dashed lines show the grounding line stillstand positions at and after 11.5 ± 0.3 cal kyr BP^8,18^.

Today, retreat rates are highly variable along the grounding line and in time^10^. The relative shortness of the satellite-era observations highlights the need to assess longer-term dynamics associated with past episodes of major ice sheet contraction. Previous studies of the Antarctic continental margin glacial morphology and sedimentology have estimated that the post-LGM retreat rates ranged between 40 and 180 ma^−1^^11–13^. Here, we evaluated a post-LGM record from the eastern Ross Sea continental shelf where several details of WAIS retreat are better constrained in the WDB. There, previous studies have demonstrated that the paleo-BIS experienced an abrupt retreat of ~ 200 km from an outer to a middle continental shelf grounding-line stillstand position (Fig. 1). Our analyses focused on the maximum rate and minimum duration for a paleo-grounding-line retreat that occurred between these two well-defined grounding-line stillstands.

### The post-LGM retreat of the BIS from the WDB outer continental shelf

The WDB is approximately 100 km wide and 250 km long between Hayes and Houtz Banks. It extends from the continental shelf edge southward below the Ross Ice Shelf calving front (Fig. 1). In north–south crossings of the basin, a broad saddle rises more than 150 m above the regional grade across the width of the basin. South of the saddle, the continental shelf is foredeepened, i.e., landward dipping, with a maximum depth of ~ 600 m.

The BIS grounding line had already retreated from its maximum (LGM) position at the continental shelf edge prior to 14.7 ± 0.4 cal kyr BP^8^. Over the next three millennia, the BIS grounding line remained within 60 km of the continental shelf edge and deposited seven grounding-zone wedges (GZWs) all of which were more than 20 m thick^8^. The combined overlapping aggradational stack of GZWs constructed the bathymetric saddle (Fig. 1b). The crest of the saddle roughly marks the stillstand extent of BIS between 14.7 ± 0.4 and 11.5 ± 0.3 cal. kyr BP.

Isopach mapping of the stillstand GZWs and chronologic control indicate that the paleo-sediment flux of BIS averaged 1.7 × 10^8^ m^3^ a^−1^ but the flux increased nearly sevenfold from 670 ± 2.0 × 10^7^ to 4.7 ± 1.0 × 10^8^ m^3^ a^−1^ following the ice shelf break up at 12.3 ± 0.6 cal kyr BP^15^. Despite ice shelf breakup, the paleo-BIS maintained its outer-continental shelf position due to the stabilizing effects of grounding-zone wedge sediment aggradation.

During the final stages of the stillstand, the ice-volume discharge exceeded the estimated average accumulation (35.6 ± 5 km^3^ a^−1^) and produced several centuries of a mass imbalance of − 45.4 ± 34 km^3^ a^−1^, equivalent to a basin-wide thinning rate of − 0.2 ± 0.14 ma^−1^^15^. Ice volume discharge exceeded the balance velocity by a factor of two and implies ice mass imbalance of − 40 Gt a^−1^ for several centuries, which eventually triggered rapid grounding line retreat at ~ 11.5 ± 0.3 cal kyr BP.

**Figure 2:**
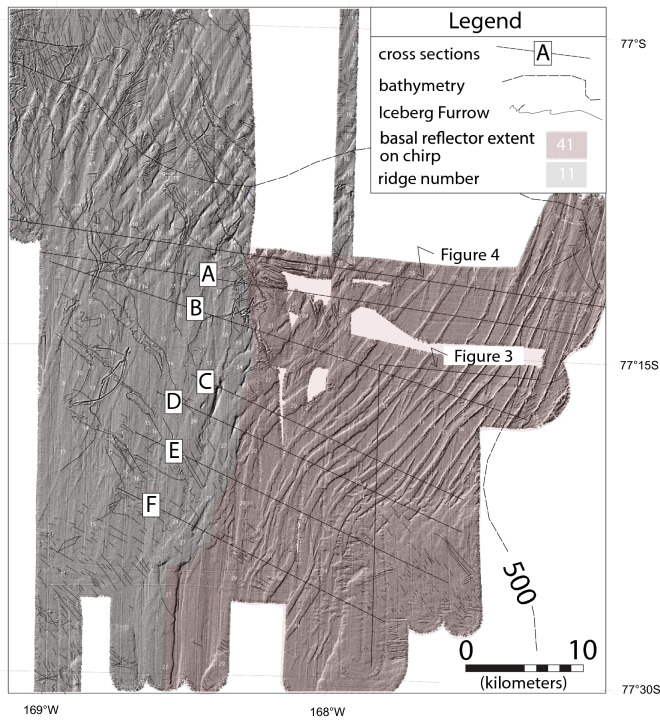
Hillshade multibeam swath bathymetry of the ridges on the middle continental shelf. The dashed line is the 500 m bathymetric contour from Davey and Nitsche^14^. The oldest ridge in the surveyed area is labeled 1. Thin black lines show the location of iceberg furrows. The shaded-area to the east demarcates where shallow-depth basal reflection is observed on chirp sonar.

**Figure 3:**
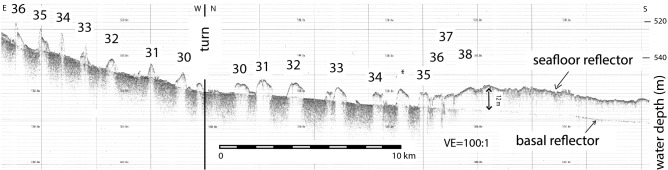
Sonar transects showing steep-flank ridges and basal reflector underlying ridges 29–50. The transect location is shown on Fig. 2.

Instead of seismically-resolvable GZWs, only small-scale moraine ridges mantle the foredeepened middle continental shelf (Figs. 2 and 3). The absence of seismically-resolvable GZWs south of the bathymetric saddle strongly suggests that there was no significant pause or re-advance as the grounding line retreated to Roosevelt Island^16,17^, which marks the site of a second post-LGM grounding-zone stillstand. The second stillstand ended at ~ 3.2 kyr BP^18^. These small sediment ridges were first noted in a previous study^19^ but the then-available data was not sufficient to recognize that they represent retreat along the eastern flank of a large evolving grounding-line embayment. The small-scale moraines on the WDB middle continental shelf are the focus of our study.

## Results

### The morainal ridges on the Whales Deep Basin middle continental shelf

A well-preserved succession of sub-parallel morainal ridges have an overall NNE to SSW trend in water depths ranging from 600 to 450 m from the central part of the WDB up along the flank of Houtz Bank (Fig. 2). The ridges extend from the crest of the bathymetric saddle to the southern end of the WDB. Several ridges have leading and trailing flanks that dip at angles up to 6° (Fig. 3). Ridge crests vary from peaked to flat topped or rounded. Some ridges pinch out along strike whereas for others, the leading and trailing edges of adjacent ridges converge and amalgamate and/or partly overlap (Fig. 2).

In the areas surveyed south of 77° 30’ S in the central and eastern side of the WDB, discontinuous crests ridges trend NNE-SSW^16^ but it is not possible to correlate these to the more continuous ridges to the north shown in Fig. 2 with the available data coverage (Fig. 1). Ridges are not observed on the western-most, dip-oriented transect of the WDB middle continental shelf^16^.

**Figure 4:**
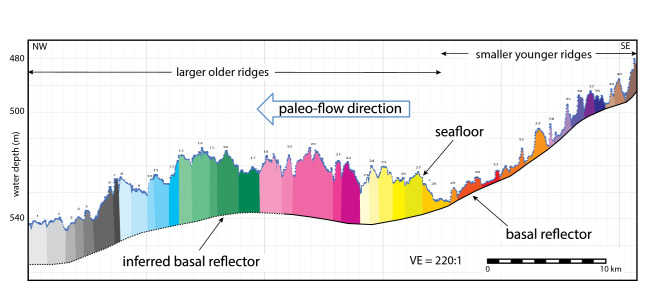
Representative NW–SE cross section of the ridge field. The basal reflection below ridges 1–28 is inferred whereas that below ridges 29–50 is from the sonar data. The colors show our interpretation of individual ridges. The location of cross section is shown on Fig. 2.

The longest individual ridges have crests that can be traced for up to 40 km. Forty ridges that extend more than 5 km are numbered where data coverage allows the stratigraphic relationships to be reasonably inferred for reference (Figs. 2, 3 and 4). Two general ridge types are recognized. Ridges 1 thru 28 are broader (averaging 1.5 km width in cross section) and have rounded crests (Figs. 3 and 4) with leading and trailing edges that mostly abut. Here, seafloor relief is low generally ranging between 4 and 10 m. Ridges 29–41 are discrete submarine landforms with less than 1 km cross sections that are mostly separated by flat-lying areas that average 1 km in width except where the ridges are amalgamated end to end. Seafloor relief for the younger ridges is generally less than 10 m.

A sharp, smoothly-varying subsurface reflection is observed on CHIRP data (Fig. 3) to mostly follow the regional bathymetric grade below ridges 19 to 41 (Fig. 4). The amplitude and continuity of the basal reflector is strongest where the overlying sediment is thin but the reflector dims significantly where the overlying sediment thickens above approximately 12 m. We infer that the basal reflector dims because of signal attenuation but continues throughout the WDB at a ~ 12-m minimum depth below the older ridges (see left hand side of Fig. 3).

### Sediment volumes of ridges on the WDB middle continental shelf

We measured the cumulative stream-wise sediment volume (measured in m^3^/m) between the seafloor and the basal reflector on six cross sections oriented approximately perpendicular to the ridge crests (Supplemental Fig. 1, Table 1). The steam-wise sediment volume reported in Table 1 is reduced by 1 m^3^/m to account for this volume of post-glacial sediment^17,20^. The stream-wise sediment volumes were converted to a depositional duration for each cross section using both the lower- and upper-end stream-wise sediment fluxes (Table 1). For the lower-end sediment flux, the cross-section sediment volumes represent only a few centuries durations of deposition as the grounding line orthogonally retreated a distance of ~ 25 km. For that measured distance and duration, the retreat rate averages 100 ± 32 ma^−1^. At the upper-end sediment flux, the same stream-wise volumes represent shorter durations of deposition spanning up to nearly five decades (Table 1). For these minimum durations, the corresponding retreat rate averages ~ 700 ± 79 ma^−1^.

**Table 1:** Stream-wise volume of sediment ridges from cross section data plus the corresponding duration and retreat rates from two end-member sediment flux estimates.

A. Cross- section name	B. Cross- section length (m)	C. Stream-wise ridge volume (m^3^/m)	D. Low-end sediment flux with buttressing (670 ± 20 m^3^/m/a)		E. High-end sediment flux without buttressing (4700 ± 100 m^3/^m/a)	
			Maximum duration (a)	Minimum retreat rate (m/a)	Minimum duration (a)	Maximum retreat Rate (m/a)
A	28,680 ± 1	185,039 ± 14,003	276 ± 78	104 ± 32	39 ± 3.9	735 ± 81
B	27,633 ± 1	153,057 ± 11,764	228 ± 66	121 ± 38	33 ± 3.3	837 ± 92
C	25,998 ± 1	163,386 ± 11,791	244 ± 66	107 ± 33	35 ± 3.5	743 ± 82
D	25,813 ± 1	207,653 ± 15,892	310 ± 89	83 ± 26	44 ± 4.4	587 ± 65
E	25,020 ± 1	221,076 ± 17,526	329 ± 98	76 ± 24	47 ± 4.7	532 ± 59
F	18,712 ± 1	102,831 ± 7906	153 ± 44	122 ± 38	22 ± 2.2	851 ± 94
Average of low- and upper-end retreat rates				102 ± 32		714 ± 79

(A) Cross section name; (B) Cross section length; (C) Stream-wise ridge volume; (D. Low-end sediment flux with ice-shelf buttressing and the inferred maximum duration and minimum retreat rate and (E) High-end sediment flux without ice-shelf buttressing and the inferred minimum duration and maximum retreat rate. The bottom row of columns D and E show the uncertainty for the low- and upper-end retreat rates averaged for cross sections A through F.

## Discussion

### Opening of the WDB embayment

The existence of a deep grounding-line embayment in the WDB was first hypothesized by Halberstadt et al.^21^. Their hypothesis was based on the orientation of megascale glacial lineations (MSGLs) in the Glomar Challenger and Little America basins that converge towards the WDB middle continental shelf (see arrows on Fig. 1b). Our mapping of the WDB ridge field confirms the embayment hypothesis and provides information as to how the opening proceeded. The location of the oldest morainal ridge shows that the embayment first opened in the deeper western side of the WDB middle continental shelf. Similarly-oriented, small-scale ridges are found even further east of our data^19^. The backstepping ridges record a succession of pauses during the retreat of the embayment’s eastern edge (Fig. 2). It is not possible to correlate any ridges across the entire WDB because of the limitations in the data coverage. Our map projections of the embayment’s western and southern flanks (Fig. 1) are mostly conjectural being based on the sparse swath-bathymetry transects and the regional bathymetry.

### Constraints on the rate and duration of grounding-line retreat at 11.5 ± 0.3 cal kyr BP

The glacial geomorphology and sedimentology of the Antarctic continental shelf provide strong constraints on former grounding-line positions and retreat^22,23^. In the WDB, the start and end chronology of the large GZW that formed during the outer continental shelf grounding-zone stillstand is well constrained^8^. Stratigraphic super-position requires that the small-scale and back-stepped morainal ridges mantling the GZW topset formed during the subsequent retreat^16^. Investigators have long assumed that such small backstepped, grounding-zone features represent short-lived intervals of deposition during retreat^24–29^. Unfortunately, the general paucity of dateable material in glacial proximal deposits on the WDB middle continental shelf makes it difficult to accurately constrain the actual rates at which grounded ice contracted. A lone foraminiferal radiocarbon date was obtained well above subglacial sediment, from within the middle of the post-glacial sediment. The stratigraphic framework and core location 10 km north of the Ross Ice Shelf calving front requires only that grounded ice had retreated from the outer continental shelf saddle to Roosevelt Island prior to 8.7 ± 0.2 cal kyr BP^8^. Other radiocarbon dates from acid insoluble organic matter on the middle shelf of the WDB are considered suspect due to a mixing of contemporaneous and old carbon^19^. More recently obtained radiocarbon dates from C14**–**C18 fatty acids only constrain the retreat of the ice shelf^20^ and thus are not relevant for the prior retreat of grounded ice.

Unfortunately, the radiocarbon dates from marine core do not constrain by when the WDB embayment had fully opened. In the absence of more radiocarbon dates, our sediment flux approach provides end-member constraints on the minimum and maximum rates of grounding-line retreat because the cumulative ridge-field volume represents grounding-zone sedimentation measured orthogonal to the embayment’s eastern flank (Fig. 4). Generally speaking, sediment yield is higher in interglacials due to more rapid flow of warmer ice^30,31^. In the WDB, the post-LGM retreat was underway prior to ~ 14.7 cal kyr BP. The low-end sediment flux we used corresponds to a part of a post-LGM grounding-zone stillstand when the BIS flow was buttressed by an ice shelf^15^. In contrast, the upper-end sediment flux we used is the average for the latter part of the outer-continental shelf stillstand following an ice shelf breakup at ~ 12.3 cal kyr BP^15^. Our end-member sediment-flux analyses constrain the WDB retreat rates to have been between ~ 100 and ~ 700 ma^−1^ (Table 1). This range is faster than most reported paleo-retreat estimates^11,12^. The fastest paleo-retreat across the Amundsen/Bellingshausen inner continental shelf averaged 180 ma^−1^^13^, which overlaps with the low-end of our WDB estimates. We dismiss the possibility that the paleo-sediment flux in the WDB was near the low end-member rate because iceberg furrows that orthogonally cross cut the sediment ridges require that full-draft icebergs calved at or near the retreating grounding zone^32^. In other words, the evidence requires either a weak to non-existent ice shelf buttressing existed as the grounding line retreated^33^, which is consistent with the high-end sediment flux estimate.

Sediment supply to the embayment’s eastern flank was probably derived from the MacAyeal Ice Stream (MIS) flowing through LAB^21^. The LAB and WDB are both underlain by Cenozoic sedimentary strata^34^ so the deglacial erosion rates would have been similar to the high end-member rate for the paleo-BIS. Moreover, the catchment for the MIS is larger than that of the BIS^35^ hence the paleo-BIS sediment flux is a reasonable proxy for that of the paleo-MIS. If we assume that the upper-end rate of ~ 700 ± 79 ma^−1^ applies to the entire grounding line retreat along the ~ 200-km centerline of the WDB (from the outer continental shelf saddle to Roosevelt Island stillstands positions), then the embayment opened in ~ 280 years.

### A paleo-perspective for the ongoing grounding-zone oscillations

From a paleo-climate perspective, the opening of the WDB embayment and associated re-organization of ice flow represented a major post-LGM collapse of the WAIS. In either of the end-member scenarios we considered, the embayment records rapid retreat that lasted from several to a few tens of centuries. These data thus provide strong support for the modeling prediction that the current rapid retreat could also be sustained for centuries^2^. It is important to recognize that the modern-day retreat of ice streams have oscillated between both faster and slower rates in time and space^36^. During the past four decades, several large ice streams in West Antarctica have exhibited rates that exceeded even the upper-end paleo-retreat rate estimate. For example, Pine Island Glacier retreated at ~ 1000 ma^−1^ for nearly a decade between 1992 and 2011^37^ but then retreat rate substantially decreased^10^. Likewise, in the Bellingshausen and Amundsen Sea sectors, several grounding zones experienced localized rapid retreat at rates ranging up to 1200 ma^-1^ between 2010 and 2016 whereas broader areas of the same regions have retreated at slower rates ranging from 100 to 300 ma^−1^^10^. Between 1990 and 2015, retreat in the Bellingshausen sector proceeded with maximum rates of ~ 200 ma^−1^ whereas adjoining drainage areas in the Amundsen-Sea sector (e.g., Smith, and Kohler glaciers) experienced faster retreat^5^. In the largest catchment of the Amundsen Sea sector, the retreat of the Thwaites Glacier increased to 420 ma^−1^ by 2016^10^. The opening of the WDB embayment may have also proceeded with similar oscillations like those observed for modern ice streams. Taken at face value, the decreased thickness of the younger ridges suggests that the retreat rate increased by ~ 70% from ~ 515 to ~ 880 ma^−1^ for that part of the embayment’s eastern flank surveyed (Supplemental Tables 1 and 2). An acceleration of ice sheet retreat on the seaward sloping bed (the western flank of Houtz Bank) is counter-intuitive but the WDB is foredeepened along its north–south axis. The similarities between the modern and paleo retreat validates the concern that today’s oscillations in several sectors of the WAIS are in the early days of an ongoing collapse that, unless re-buttressed, could be sustained for centuries to come. Our data cannot be used to infer whether either melting by warm waters or other factors such as subglacial geology^38^ controlled the paleo-retreat. Nonethless, the comparison suggests that it is important for policy makers to consider the impacts of a sustained WAIS contraction.

## Methods

We integrated the regional seismic stratigraphy^39^ with a new, detailed analysis of multibeam swath bathymetry survey and chirp profiles, plus extracted cross sections to reconstruct the pattern of the grounding line retreat from the WDB middle continental shelf. The multibeam data were collected with a hull-mounted Kongsberg EM122 swath bathymetric system from the *Nathaniel B. Palmer RVIB* during expedition NBP1502B. The EM122 operates at 12 kHz and records up to 432 beams. The maximum port- and starboard side angle is 75°. Time-depth corrections were performed from expendable bathythermographs that were taken at least once every 24 h. The initial multibeam was corrected and processed onboard with Teledyne CARIS HIPS and SIPS using a final CUBE function to create a 20 × 20 m resolution DEM raster. The multibeam DEM was then imported to QGIS 3.18 for geospatial interpretation, and calculation. Sub-bottom sonar was continuously co-acquired with the multibeam with a hull-mounted Knudsen 3260 3.5 kHz Chirp echosounder operated with a 64 ms sweep around 3.5 kHz, which provides a vertical resolution of 0.1 m and horizontal resolution of 10 m for a water depth of 500 m.

We analyzed the morainal-ridge dimensions, orientations, morphology and volume with ArcGIS and ArcMap software. The multibeam bathymetric survey was interpreted in ArcMap 10.7 as a raster datasheet. A prominent reflector is observed on CHIRP data to underlie the ridge field over approximately half of the area of interest.

A basal reflector observed to underlie part of the surveyed area on CHIRP data was correlated and mapped in Petrel 18.1. An initial subsurface map of the basal reflector was converted to meters depth below sea level with a velocity of 1650 ms^−1^. This surface was then exported as a series of point features and interpolated using an Intermediate Distance Weighting (IDW) interpolation within QGIS 3.18 creating a final DEM of the basal surface. Cross-sections were generated using the Line Interpretation Tool in the 3D Analysis Toolbox 7 on a common vertical and horizontal scale.

In QGIS 3.18, we clipped the seafloor DEM to the extent of the basal reflector DEM so that Raster calculations could be computed to the edges of that data set. The clipped seafloor DEM was subtracted from the basal reflector DEM to create an isopach of positive height between the basal reflector and seafloor.

Cross-sections across the isopach have x values corresponding to distance and y-values corresponding to height of sediment above the basal surface. The y-positions (vertical measure of sediment thickness between the seafloor and the basal reflector) were multiplied by the individual spacing between x positions and the areas were summed to estimate cross-sectional area of the ridge field moraines. We converted the cross-sectional areas to a stream-wise volume by multiplying the cross-section area by 1 m of the paleo-grounding line length measured perpendicular to the ridge crest.

We converted the lower- and upper-end BIS paleo sediment fluxes from Bart and Tulaczyk^15^ (2020) to a stream-wise quantity (in m^3^/m/a) by dividing their 3D flux value by 100,000 m (i.e., the width of the paleo-BIS). The lower-end sediment flux (670 ± 20 m^3^/m/a) corresponds to the average rate of grounding-zone deposition prior to ice shelf breakup (between 14.7 ± 0.4 and 12.3 ± 0.6 cal kyr BP). The upper-end sediment flux (4700 ± 100 m^3^/m/a) corresponds to the average rate sediment after the ice-shelf breakup (between 12.3 ± 0.6 and 11.5 ± 0.3 cal kyr BP). We used the stream-wise fluxes to estimate the elapsed time to deposit the morainal ridges along cross sections. The time elapsed to deposit the ridges is the quotient of a cross-section’s cumulative sediment volume and the paleo-BIS stream-wise sediment flux. We then estimated the grounding-line retreat rate by dividing the cross-section distance (from the oldest to the youngest ridge) by the time elapsed to deposit the ridges.

## Supplementary Information

Supplementary Information.

## Data Availability

The NBP1502B expedition data reported in the article is archived on the Marine Geoscience Data System (https://www.marine-geo.org/tools/search/entry.php?id=NBP1502).
