# Supplementary material for: A paleo-perspective on West Antarctic Ice Sheet retreat
Source: Sci Rep. 2022 Oct 21;12:17693. doi: 10.1038/s41598-022-22450-3 (PMC9586952; doi:10.1038/s41598-022-22450-3)
Supplement: Supplementary file 1 — Supplementary Information. [file 41598_2022_22450_MOESM1_ESM.pdf]

A paleo-perspective on West Antarctic Ice Sheet retreat

Philip J. Bart[1*]

Matthew Kratochvil[1]

[1] Louisiana State University, Department of Geology and Geophysics, Baton Rouge, Louisiana 70803, USA

* Corresponding author (pbart@lsu.edu)

Supplemental Figure 1. Ridge Field Cross Sections A through F.
Cross sections show the Whales Deep Basin ridges with respect to sea level. The locations of the cross sections are shown in Supplemental Figures 2 and 3. The basal reflector is an interpolation derived from chirp sonar. The interpolation produced minor areas of cross over between the seafloor and the basal reflector (shown in gray shade) that were manually corrected.

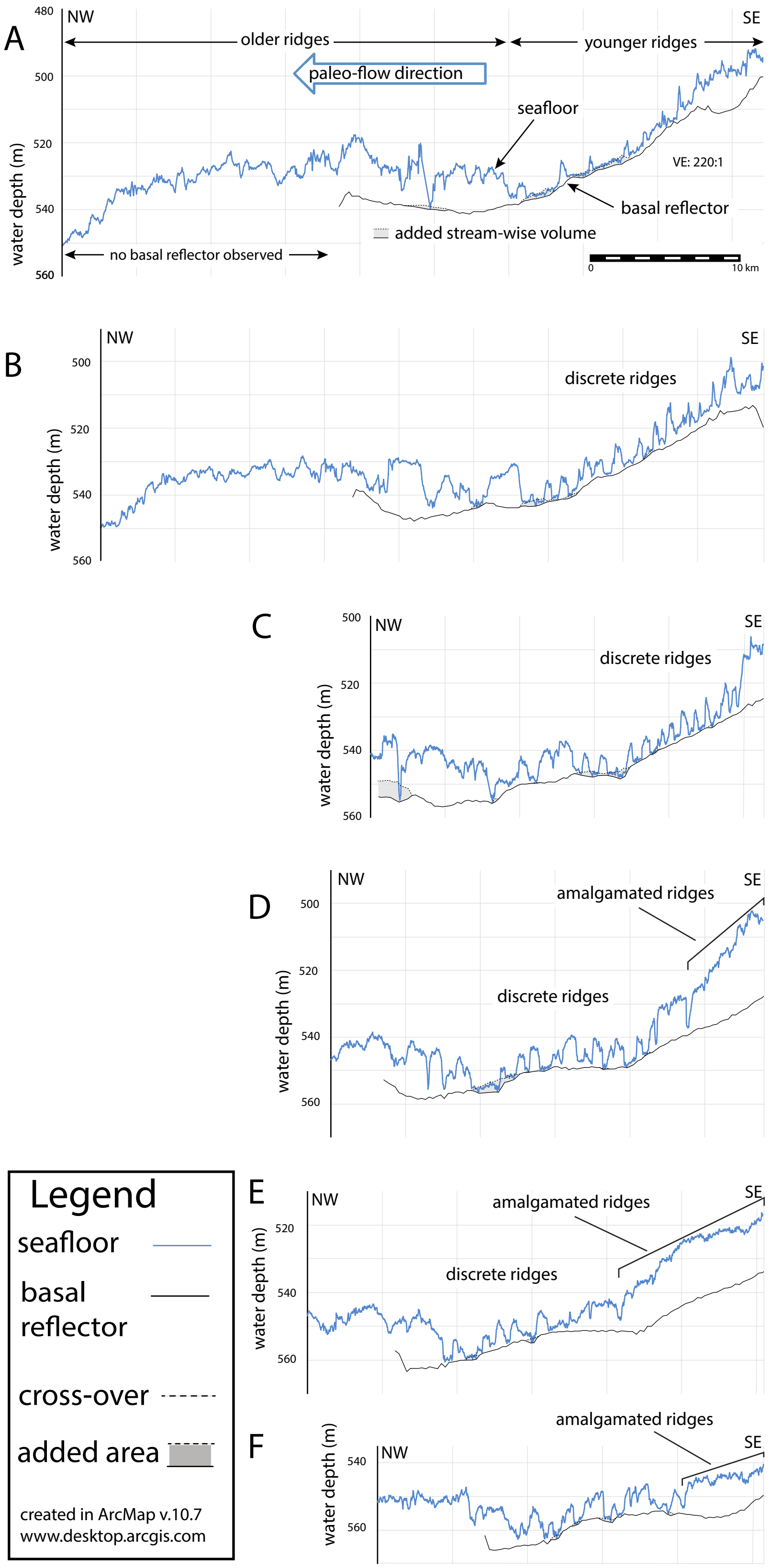

Supplemental Figure 2. Contour map of the Basal Reflector.
The map shows elevation of the basal refector underlying the Whales Deep Basin Ridge field. The depths are reported with respect to sea level. The locations of cross sections A through F (see Supplemental Figure 1) are shown.

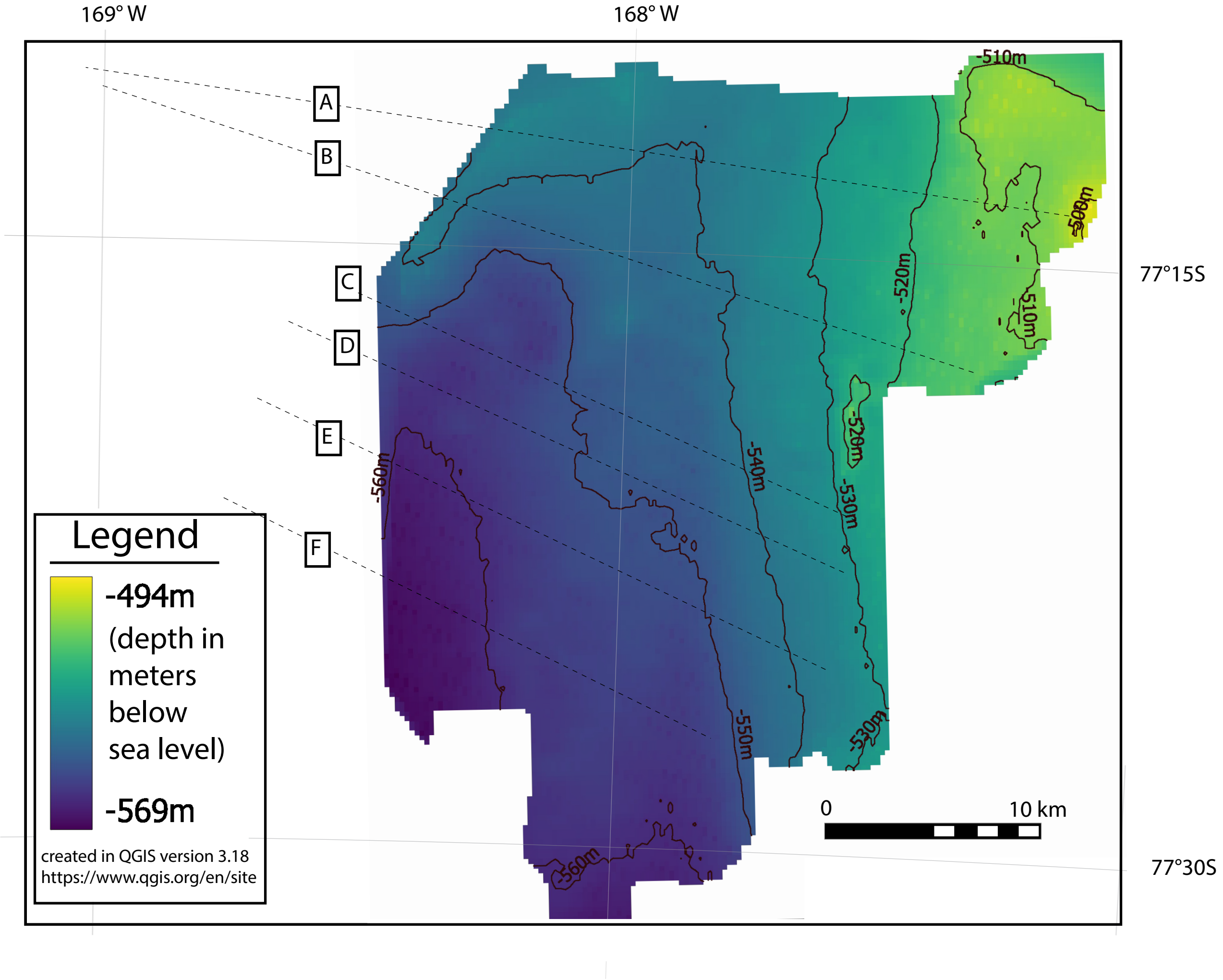

Supplemental Figure 3. Isopach (thickness) map of the ridge field.
The maps shows a color-coded thickness of the Whales Deep Basin ridge field in meters.
The locations of cross sections A through F (see Supplemental Figure 1) are shown.

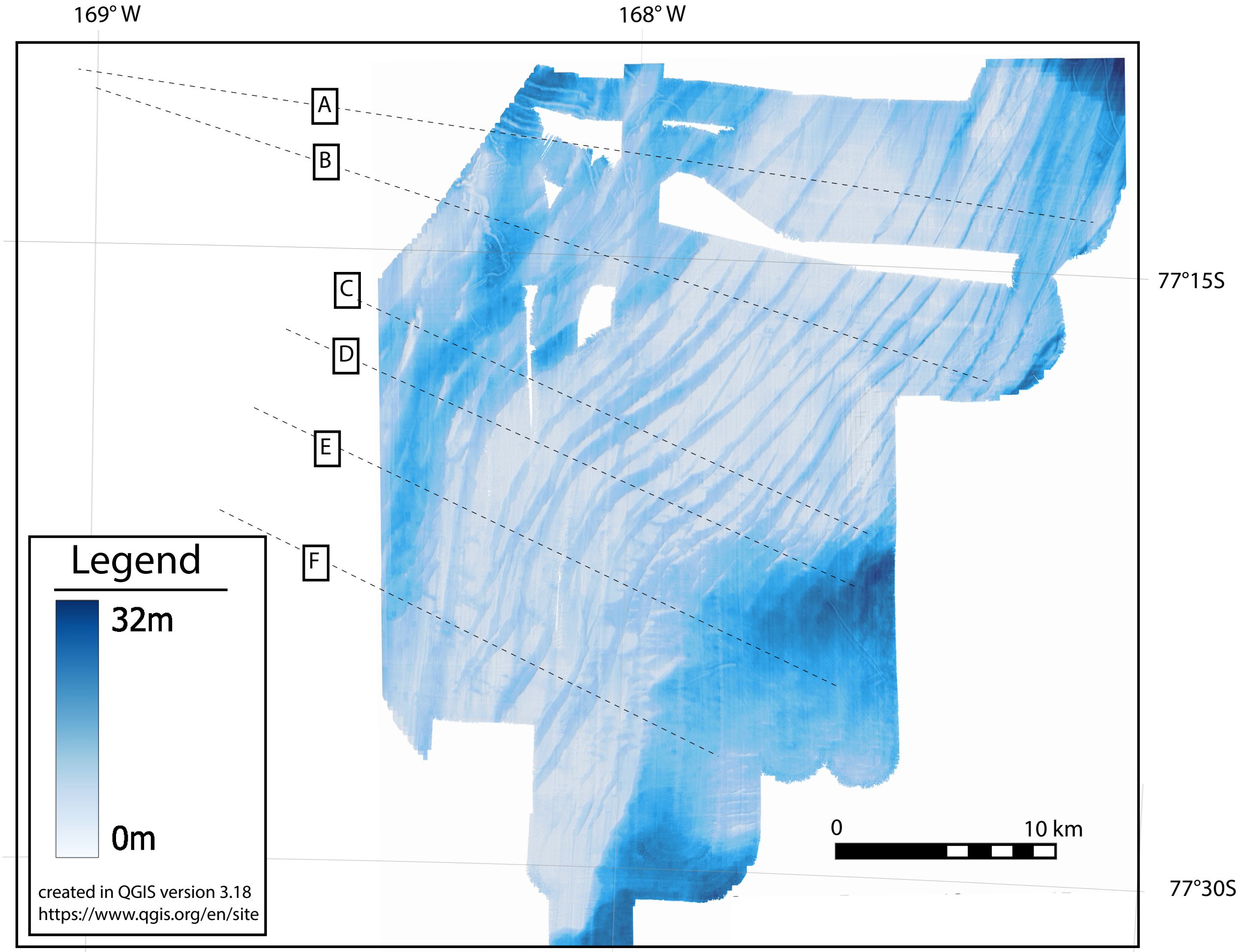

Supplemental Table 1. Cross-section data for older ridges (i.e., 19 through 28). A) Cross section name; B) Cross section length; C) Stream-wise volume; D) Low-end sediment flux with ice-shelf buttressing and the associated maximum duration and minimum retreat rate; E) High-end sediment flux without ice-shelf buttressing and the associated minimum duration and maximum retreat rate. The bottom row of columns D and E show the uncertainty for the low- and upper-end retreat rates averaged for cross sections A through F.

A. Cross section name	B. Cross section length for older-ridges (m)	C. Stream-wise volume of older ridges (m^3/m)	D. Low-end sediment flux with buttressing (670 ± 20 $m^3/m/a$)		E. High-end sediment flux without buttressing (4700 ± 100 $m^{3/}m/a$)	
			Maximum Duration (a)	Minimum Retreat rate (m/a)	Minimum Duration (a) (10%)	Maximum Retreat Rate (m/a)
A	12103 ± 1	124707 ± 9817	186 ± 56	65 ± 20	27 ± 2.9	448 ± 41
B	8663 ± 1	66987 ± 5279	100 ± 30	87 ± 27	14 ± 1.5	619 ± 68
C	3082 ± 1	37835 ± 2147	56 ± 17	55 ± 24	8 ± 0.9	385 ± 39
D	1380 ± 1	12053 ± 964	18 ± 5	77 ± 24	2.6 ± 0.3	538 ± 59
E	711 ± 1	6892 ± 551	10 ± 3	71 ± 22	1.5 ± 0.2	485 ± 53
F	2908 ± 1	20859 ± 1669	31 ± 9	94 ± 29	4.4 ± 0.5	665 ± 73
low- & upper-end retreat rates for ridges 19 thru 28				75 ± 22		515 ± 57

Supplemental Table 2. Cross section data for younger ridges (i.e., 29-41). A) Cross section name; B) Cross section length for younger ridges; C) Stream-wise volume of younger ridges; D) Low-end sediment flux with ice-shelf buttressing and the associated maximum duration and minimum retreat rate for younger ridges; E) High-end sediment flux without ice-shelf buttressing and the associated minimum duration and maximum retreat rate for younger ridges. The bottom row of columns D and E show the uncertainty for the low- and upper-end retreat rates averaged for cross sections A through F.

A. Cross section name	B. Cross section length (m)	C. Stream-wise volume of younger ridges (m^3/m)	D. Low-end sediment flux with buttressing (670 ± 20 $m^3/m/a$)		E. High-end sediment flux without buttressing (4700 ± 100 $m^{3/}m/a$)	
			Maximum Duration (a)	Minimum Retreat rate (m/a)	Minimum Duration (a)	Maximum Retreat Rate (m/a)
A	16577 ± 1	60718 ± 4217	91 ± 27	182 ± 55	13 ± 1.3	1275 ± 140
B	18970 ± 1	79070 ± 5925	118 ± 35	161 ± 48	17 ± 17	1116 ± 123
C	22916 ± 1	125551 ± 9644	187 ± 56	123 ± 37	27 ± 27	849 ± 93
D	24443 ± 1	195600 ± 14928	292 ± 88	84 ± 25	42 ± 4.2	582 ± 64
E	24309 ± 1	214183 ± 16975	320 ± 96	76 ± 23	46 ± 4.6	528 ± 58
F	15803 ± 1	80793 ± 6223	121 ± 36	131 ± 40	17 ± 1.7	930 ± 102
low- & upper-end retreat rates for ridges 29 thru 41				126 ± 38		880 ± 97
